# Predictive Model for the Non-Invasive Diagnosis of Endometriosis Based on Clinical Parameters

**DOI:** 10.3390/jcm12134231

**Published:** 2023-06-23

**Authors:** Lutz Konrad, Lea M. Fruhmann Berger, Veronica Maier, Fabian Horné, Laura M. Neuheisel, Elisa V. Laucks, Muhammad A. Riaz, Frank Oehmke, Ivo Meinhold-Heerlein, Felix Zeppernick

**Affiliations:** Institute of Gynecology and Obstetrics, Faculty of Medicine, Justus Liebig University Giessen, 35392 Giessen, Germany; lea.fruhmann@hotmail.com (L.M.F.B.); veronica.maier@t-online.de (V.M.); fabian.horne@gmail.com (F.H.); laura.m.neuheisel@med.uni-giessen.de (L.M.N.); elisa.v.laucks@med.uni-giessen.de (E.V.L.); muhammad.a.riaz@gyn.med.uni-giessen.de (M.A.R.); frank.oehmke@gyn.med.uni-giessen.de (F.O.); ivo.meinhold-heerlein@gyn.med.uni-giessen.de (I.M.-H.); felix.zeppernick@gyn.med.uni-giessen.de (F.Z.)

**Keywords:** endometriosis, pelvic pain, neuropathic pain, PainDETECT, prediction model, pre-operative diagnosis, questionnaire

## Abstract

Objectives: Are other pain symptoms in addition to dysmenorrhea, dyspareunia, dyschezia, dysuria, and chronic pelvic pain correlated to endometriosis and suitable for a clinical prediction model? Methods: We conducted a prospective study from 2016 to 2022, including a total of 269 women with numerous pain symptoms and other parameters. All women filled out two questionnaires and were examined by palpation and transvaginal ultrasound (TVUS). In cases of suspected deep endometriosis, magnetic resonance imaging (MRI) was performed. After the operation, endometriosis was diagnosed by histological examination. Results: All in all, 30 significant parameters and 6 significant numeric rating scale (NRS) scores associated with endometriosis could be identified: 7 pain adjectives, 8 endometriosis-associated pain symptoms, 5 pain localizations, 6 parameters from the PainDETECT, consumption of analgesics, and allergies. Furthermore, longer pain duration (before, during, and after menstruation) was observed in women with endometriosis compared to women without endometriosis (34.0% vs. 12.3%, respectively). Although no specific pain for endometriosis could be identified for all women, a subgroup with endometriosis reported radiating pain to the thighs/legs in contrast to a lower number of women without endometriosis (33.9% vs. 15.2%, respectively). Furthermore, a subgroup of women with endometriosis suffered from dysuria compared to patients without endometriosis (32.2% vs. 4.3%, respectively). Remarkably, the numbers of significant parameters were significantly higher in women with endometriosis compared to women without endometriosis (14.10 ± 4.2 vs. 7.75 ± 5.8, respectively). A decision tree was developed, resulting in 0.904 sensitivity, 0.750 specificity, 0.874 positive predictive values (PPV), 0.802 negative predictive values (NPV), 28.235 odds ratio (OR), and 4.423 relative risks (RR). The PPV of 0.874 is comparable to the positive prediction of endometriosis by the clinicians of 0.86 (177/205). Conclusions: The presented predictive model will enable a non-invasive diagnosis of endometriosis and can also be used by both patients and clinicians for surveillance of the disease before and after surgery. In cases of positivety, as evaluated by the questionnaire, patients can then seek advice again. Similarly, patients without an operation but with medical therapy can be monitored with the questionnaire.

## 1. Introduction

Endometriosis is characterized by the implantation and growth of endometrial glands and stroma outside the uterine cavity [1], with pain and infertility as the main symptoms of patients [2]. Pain is an unpleasant sensory and emotional experience associated with actual or potential tissue damage or described in terms of such damage [3]. 

Dysmenorrhea, chronic pelvic pain (CPP), chronic non-menstrual pelvic pain, and dyspareunia are the most consistently reported pain types [2], with dysmenorrhea as the most frequent pain symptom and the highest pain perception among women with endometriosis [4,5]. Women with proven endometriosis reported the highest chronic/cyclic pain and significantly greater dyspareunia, dysmenorrhea, and dyschezia compared to women with other gynecologic pathologies or a normal pelvis [6]. Pelvic pain associated with primary dysmenorrhea typically occurs with the onset of menstruation and lasts for 8–72 h [2]. Data are inconsistent regarding a link of pain characteristics to the location or staging of endometriosis [2,6]; however, the total number of ectopic endometrial implants seems to be nevertheless associated with the intensity of dysmenorrhea [7]. 

A higher likelihood of suffering from endometriosis was found with an increased number of symptoms present (5-fold for one symptom; 85-fold ≥ 7 symptoms) [8]. A large study based upon the endometriosis health profile (EHP)-30 questionnaire revealed more menstrual pain (~4-fold), abdominal pain unrelated to menses (~6.5-fold), defecation pain (~6-fold), irregular bleeding (~4-fold), and bowel irritation such as constipation/diarrhea (~4,6-fold) in women with endometriosis compared to healthy women [9]. Another questionnaire study identified dysmenorrhea, dyspareunia, dysuria, lower back pain, pelvic pain other than during menses, rectal pain, and pain at ovulation or intercourse as significantly associated with endometriosis [10]. Furthermore, a positive correlation was found between dysmenorrhea and chronic pelvic pain (CPP), between dysmenorrhea and dyspareunia, and between constipation and dysuria. A study with patient-reported outcomes (n = 107) using a 36-item checklist for the symptoms identified very high pain values: dysmenorrhea (94.4%), dyspareunia (70.1%), infertility (63.6%), dyschezia (44.9%), and dysuria (22.4%) in endometriosis patients [11]. However, the use or nonuse of contraceptives was not reported. An online survey with several distinct questionnaires identified menstrual pain severity and duration, bloating, nausea, and widespread pain sites as significant predictors of endometriosis [12]. 

No single pain parameter reached significance as a prognostic factor, but a constellation of endometriosis-related symptoms seems to be a strong predictor of the disease [2]. 

The use of the short-form McGill questionnaire showed cramping as a significant pain parameter, resulting in a sensitivity of 0.92, a specificity of 0.33, a PPV of 0.40, and an NPV of 0.89 [13]. However, other significant pain parameters, such as throbbing, gnawing, and dragging pain to the legs were not found [14]. A questionnaire based upon 8 modules and 47 questions retained four significant variables, namely CPP, dyspareunia ≥ 3, painful defecation, and acne, with a final predictive logistic model for endometriosis with high sensitivity (0.902) and specificity (0.750) [15].

Up to 50% of women who experience infertility have endometriosis [16,17]. The population of infertile women with endometriosis is heterogenous, and diverse phenotypes can be observed in the clinical setting. The causes of infertility due to endometriosis are multiple and include reduced ovarian reserve, pain during sex resulting in reduced frequencies of intercourse, but also adenomyosis, uterine fibroids, and especially deep infiltrating endometriosis (DIE) [16,17]. A recent review stressed the importance of surgery in the case of DIE to improve fertility outcomes [17].

In recent years, neuropathic pain associated with endometriosis has also come into focus and has been found to have a high frequency of 40% [18]. Neuropathic pain is characterized by burning pain, evoked pain, abnormal temporal summation, hyperalgesia, and allodynia and does not, or only to a small extent respond to common analgesics [19,20]. The distinction between neuropathic pain and nociceptive pain might be challenging; however, both contribute to chronic pain [19]. 

Up to date, the preoperative use of questionnaires and predictive models in the diagnosis of all types of endometriosis is scarce. Thus, we investigated endometriosis-associated pain with two questionnaires and identified 30 significant parameters and 6 significant NRS scores. A decision tree model resulted in high predictive values, suggesting that a questionnaire might be an attractive tool for the preoperative diagnosis of all types of endometriosis.

## 2. Material and Methods

A questionnaire (in German) was developed to identify general features and determine different aspects of pain (Figure S1): (i) general features such as body mass index (BMI), age, cycle length, endometriosis in relatives, allergies, fertility/parity, etc. (ii) “classical” descriptions of pain such as dysmenorrhea, dyschezia, dysuria, chronic pelvic pain, constipation, and fertility, mostly in combination with the intensity of the pain (NRS 0–10); (iii) different pain descriptors such as cramping, tearing, pulling, stinging, pulsatile, touch-sensitivity, burning, pressing, diffuse, heat, flashing, and [iv] the PainDETECT questionnaire for the investigation of neuropathic pain [21,22]. The PainDETECT questionnaire can be accessed at www.pfizerpcoa.com in different languages, including English (last access: 12 December 2022).

Many of the pain descriptors used in the present study were derived from data collected in a previous study with a structured questionnaire [23]. The extended and reorganized questionnaire of the present study was based upon an extensive literature review for pain descriptors for endometriosis, the experiences of clinicians, and more importantly, the experiences of women suffering from pelvic pain and endometriosis.

Pain areas were identified from the markings of patients on an anatomical figure given in the PainDETECT questionnaire and summarized for statistical analysis into the lower abdomen, lumbar spine, thighs/lower extremities, hips/groins, upper abdomen, vagina/mons pubis, and gluteal region. Pain intensities were measured on an NRS 11-point scale from 0 to 10, with 0 indicating no pain and 10 indicating the worst pain. Allergies included the following items: analgesics, antibiotics, band-aids, nickel and other contact agents, hay fever, food, and one gap where the patients could fill in other problems with allergies.

The questionnaires were handed out to the patients before the clinical evaluation. Health professionals and/or medical students helped the patients fill out both questionnaires. The completed questionnaires, clinical examinations, surgical findings, and histological diagnoses were collected by the researchers for data analysis. The data were anonymized and tabulated in Excel.

The prospective study was conducted at the University Hospital in Giessen, Germany. The study protocol was approved by the local ethics committee (No. 95/09, July 2009), and informed consent was obtained from all patients. We used the following inclusion criteria: all women with pelvic and infertility problems and all women who have been transferred to our endometriosis center by established doctors. We used the following exclusion criteria: patients suffering from cancer, pregnant women, women with a pelvic laparoscopy at least 6 months before visiting our center, women with bladder infections, and women suffering from nutcracker syndrome. 

All women were examined by physical examination, palpation, and TVUS to exclude endometriosis. In cases of suspected deep endometriosis, an MRI was performed. Only patients suffering from pain, especially dysmenorrhea, failed medical therapy, or infertility were operated on and subsequently histologically examined. Deep infiltrating endometriosis was classified intraoperatively by the ENZIAN score [24].

### Statistics

The validity of the questionnaire was tested with scale reliability testing using Cronbach’s alpha, which resulted in a 0.736, indicating good reliability. Furthermore, content validity testing using the scale content validity index/average (S-CVI/Ave) with 8 raters resulted in a score of 0.926, which is above the threshold value of 0.78 for 6–8 raters [25].

The decision tree was created with XLSTAT for Excel using the classification and regression tree (CART) method with the following settings: all qualitative and quantitative significant parameters were identified except for fertility because of too many nulliparous patients; maximal tree depth of 9 complexity parameter (CP) of 0.0001; and for missing data, the means were used. To overcome the problem of overfitting the data, we examined each branch and reduced or replaced it when necessary. Simpler branches that yielded results similar to those replaced were favored by the experts and investigated further. 

Each parameter was evaluated with 2 × 2 contingency tables and Fisher’s exact test to calculate NPV, PPV, sensitivity, specificity, OR, and RR. Furthermore, the outcome of the decision tree was examined for NPV, PPV, sensitivity, specificity, etc. For NRS scales, the means ± SDs were calculated and evaluated with the non-parametric test of Mann–Whitney. The calculation of cut-offs was done with a receiver operating characteristics (ROC) curve and area under the curve (AUC) with a 95% confidence interval. *p* values ≤ 0.05 were considered statistically significant. 

## 3. Results

In our questionnaire, 49 main items and 5 NRS scores were asked, and in PainDETECT, 13 items and 7 NRS scores were asked. On average, it took the patients ~15–20 min to fill out both questionnaires. We present only an extract with the most interesting items because some questions were not answered sufficiently or the patients could not remember them adequately. This study included a total of 690 women with several pelvic problems, mostly period pain. 434 women were excluded from the study because of contraception and inadequate questionnaire data, leaving a total of 269 patients for evaluation (Table 1). The patients using contraception have been evaluated separately, and the results will be published elsewhere. 

The diagnosis of endometriosis by histological examination was possible in 99% (175/177) of women with endometriosis; in two women, the operation showed lesions that could not be excised. Endometriosis could not be confirmed in 28 operated-on cases. In total, we found 11 ovarian, 7 ovarian/superficial, 33 ovarian/deep infiltrating, 31 superficial, 25 superficial/deep infiltrating, 51 deep infiltrating, and 17 ovarian/superficial/deep infiltrating endometriosis cases. One case presented with ovarian and pneumothorax, and the other with deep infiltrating and paracolic endometriosis lesions. 

### 3.1. General Parameters

No correlations except for allergies could be found for age, BMI, smoking habits, and age at menarche with or without endometriosis (Table 1). A higher percentage of women (~3-fold) with endometriosis compared to women without endometriosis were infertile (33.5% vs. 12.4%), but the number of abortions was not different between both groups (Table 1). However, it should be kept in mind that in both groups, a high proportion of women were nulliparous. 

Both groups did not differ significantly with respect to differences in cycle length, cycle regularity, or bleeding strength during menstruation (Table 1). Although women in both groups reported the intake of analgesics mainly during menstruation, a significantly higher proportion of women with endometriosis (80.8% vs. 51.1%, endometriosis (EM) cases vs. controls) used them (Table 1). Remarkably, we identified a higher number of EM cases (34.4%) compared to women without endometriosis (15.8%) who took analgesics for a longer time, from the very beginning of menses and during menses (Table 1).

### 3.2. Dysmenorrhea

The majority of both patient groups suffered from dysmenorrhea; however, the frequency and severity were significantly different (Table 2). Most of the women with endometriosis experienced dysmenorrhea (172/177 = 97.2%) compared to women without the disease (68/92 = 73.9%; Table 2). A cutoff at NRS ≤ 3 was even better (Table 2). Furthermore, pain severity was significantly higher in women with endometriosis (7.8 ± 2.2) compared to women without endometriosis (5.1 ± 3.8). Remarkably, women without endometriosis experienced significantly shorter pain duration during menstruation (32.8%) and less long pain duration before/during/after menstruation (11.9%) compared to women with endometriosis (during, 17.2%; before/during/after, 39.9%). In summary, more women with endometriosis showed longer pain duration during menstruation compared to women without the disease (Table 2). 

### 3.3. CPP and Dysuria (Painful Urination)

A significantly higher number of patients with endometriosis suffered from CPP compared to controls (82/177 = 46.3% vs. 19/72 = 20.7%; Table 2). Pain severity was ~2-fold higher in cases with endometriosis (NRS 2.9 vs. 1.4). Remarkably, a very clear difference could be identified between both groups with respect to dysuria: significantly more women with endometriosis (57/177 = 32.2%) suffered from painful urination compared to a very low number of women without endometriosis (4/92 = 4.3%). Furthermore, pain severity was also clearly different (~7.5-fold) between both groups (1.63 vs. 0.22, EM cases vs. controls, Table 2).

### 3.4. Dyschezia (Painful Defecation), Obstipation, and Diarrhea

A much larger proportion of women with endometriosis reported pain associated with defecation compared to women without endometriosis (83/177 = 46.9% vs. 14/92 = 15.2%, Table 2). Additionally, a ~3.5-fold higher NRS score for the severity of pain during defecation in cases with endometriosis compared to cases without endometriosis could be identified (Table 2). Similarly, a significantly higher number of women with endometriosis compared to women without the disease experienced obstipation (71/177 = 40.1% vs. 14/92 = 15.2%) and diarrhea (63/177 = 35.6% vs. 19/92 = 20.7%; Table 2). 

### 3.5. Dyspareunia (Painful Intercourse)

The proportion of women with endometriosis reporting pain during intercourse was clearly higher (104/177 = 58.8% vs. 26/92 = 28.3%), and they also suffered from a ~2-fold higher NRS score (3.5 ± 3.4) compared to women without the disease (1.5 ± 2.6). An NRS cutoff of ≤1 slightly improved the discrimination between both groups (Table 2).

### 3.6. Other Pain Parameters

Cramping, pulling, tearing, stinging, pulsatile burning, and touch sensitivity all showed a significant correlation with endometriosis, with the highest values for cramping, pulling, tearing, and stinging, suggesting that the main pain adjectives for endometriosis might denote primarily mechanical pain (Table 3).

### 3.7. PainDETECT and Pain Localization

In order to analyze the correlation of neuropathic pain to endometriosis, we used the PainDETECT questionnaire, originally developed for the detection of neuropathic pain caused by back pain [21,22]. 

All three pain scores—current pain, strongest pain, and average pain—during the last 4 weeks showed significant associations with endometriosis, both in the proportions of patients as well as pain intensity (Table 4). Pain was localized by a significantly higher proportion of patients with endometriosis in the lower back, lower abdomen, thighs/lower extremities, and hips/groins compared to cases without endometriosis (Table 4). A near-significant trend could also be observed for pain localized in the upper abdomen (*p* = 0.055, Table 4).

Remarkably, the pain course pattern was also different. More women with endometriosis (~3-fold) experienced regular pain attacks with pain between them (16.6%) compared to women without endometriosis (5.8%, Table 4). 

The final score of the PainDETECT questionnaire classifies the patients with respect to neuropathic pain. The proportion of women with endometriosis who probably suffered from neuropathic pain (4.2%, final score ≥ 19) was nearly similar to the proportion of women without endometriosis (1.1%), thus suggesting that neuropathic pain is not a major pain parameter associated with endometriosis (Table 4). However, with a cutoff ≤ 3, we identified a significantly higher final score in patients with endometriosis (8.5 ± 5.5) compared to women without endometriosis (3.7 ± 4.8; Table 4).

### 3.8. Decision Tree and Prediction of Endometriosis

**Figure 1:**
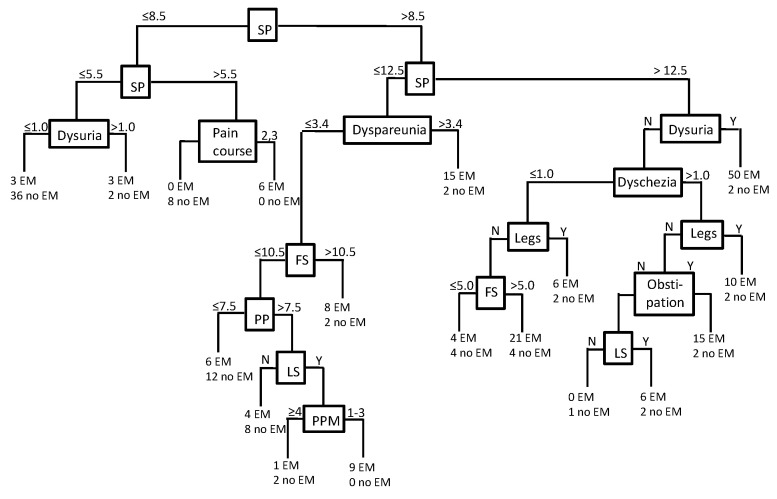
The number of significant parameters (SP) with a cutoff of 8.5 could be used as the root of the decision tree. In summary, 11 significant parameters were useful for the prediction. From the PainDETECT questionnaire, we used the final score (FS), the pain course pattern, pain radiating to the legs (legs), and lumbar spine pain (LS). The “classical” endometriosis pain parameters (dysuria, dyschezia, dyspareunia, period pain (PP), and obstipation) gave good discrimination. Furthermore, the duration of period pain during menstruation (PPM) was useful as a parameter.

All in all, 30 significant parameters and 5 NRS scores were associated with endometriosis in the present study (Table 1, Table 2, Table 3 and Table 4); however, infertility and abortion were not considered for further analysis due to the very high number of nulliparous women in both groups (Table 1). Similarly, for some parameters such as current pain, mean pain, strongest pain, etc. (Table 1, Table 2, Table 3 and Table 4), no meaningful cutoffs could be determined; therefore, only the presence or absence of these parameters was evaluated in the present study. Women with endometriosis experienced a higher number of significant parameters (14.1 ± 4.2) compared to women without endometriosis (7.8 ± 5.8). A cutoff of ≤8 resulted in fair discrimination between women with and without endometriosis (Table 5). However, because the specificity was only moderate (0.50, Table 5), we decided to construct a decision tree (Figure 1) with high *p* values and a good distinction between women with and without endometriosis (Table 5). The PPV of 0.874 is comparable to the positive prediction of endometriosis by the clinicians of 0.86 (177/205).

## 4. Discussion

In our study on the pain typology of endometriosis, we analyzed a very large number of parameters. Thus, we suggest that no single parameter but instead pain patterns are highly predictive for endometriosis. In contrast to women without endometriosis, women with endometriosis do not have different pain but experience more pain (~2-fold). A subgroup suffered from dysuria; a ~4-fold higher number experienced longer menstrual pain duration; and ~2-fold more patients suffered from pain radiating to the thighs/legs. Remarkably, a very high number of cases described their pain as cramping, tearing, or pulling, which points to the uterus as the main pain contributor. It was somewhat surprising that neuropathic pain was not very common in women with endometriosis compared to women without endometriosis. Based upon the significant parameters, a decision tree was developed, resulting in 0.904 sensitivity, 0.750 specificity, 28.235 OR, and 4.423 RR, suggesting that questionnaires, as used in this study, might be an attractive tool for pre-operative and non-invasive endometriosis diagnosis.

Pain and sub-/infertility are the most troublesome symptoms for women with endometriosis [26]. We identified significantly higher values in women with endometriosis compared to women without endometriosis (49.6% vs. 20.8%, respectively), which was also described by others (11.6% vs. 3.4%, respectively) [27]. Interestingly, sub-/infertility does not seem to be based upon a higher number of abortions in endometriosis cases because we, Sinaii et al. [26] and Ricci et al. [15], did not find differences compared to cases without endometriosis. No associations except for allergies could be identified in our study for age, BMI, smoking, menstruation length, cycle duration, abnormal menses, or irregular cycles, which have been reported with controversial data [15,28,29,30,31]. 

A significantly higher proportion of women with endometriosis (80.8%) used analgesics for a longer period of time during menses compared to women without endometriosis (51.1%), similar to a previous report [15]. However, they did not differentiate between the use and nonuse of hormonal contraception. 

Analysis of dysmenorrhea, CPP, dysuria, dyschezia, dyspareunia, obstipation, and diarrhea resulted in a highly significant association with endometriosis comparable to recent observations [2,11]. All classical endometriosis parameters showed significantly higher NRS scores in women with endometriosis compared to cases without endometriosis. Remarkably, an inverse relationship was identified for the first time in women with endometriosis compared to women without endometriosis for pain duration during menses (32.8% vs. 17.2%, respectively) and pain before/during/after menses (11.9% vs. 39.9%, respectively).

Interestingly, ~7.5-fold more women with endometriosis (32.2%) suffered from dysuria compared to only very few women without endometriosis (4.3%), similar to the study by Ricci et al. [15], but who did not discriminate between use and nonuse of contraception. Another study, but without a control group, also identified dysuria in a high number (22.4%) of endometriosis patients [11]. Due to the highly discriminative nature of dysuria in a subgroup of patients, this parameter was used for our prediction model. Thus, it is important to make sure that pain during urination is not caused by a bladder infection. 

Evaluation of pain with adjectives in our study revealed that a significantly larger proportion of women with endometriosis described their pain as cramping, tearing, pulling, stinging, pulsatile, burning, and touch-sensitivity compared to women without endometriosis. Another study also identified cramping as a significant pain parameter for endometriosis [13]. Similarly, more women with (vs. without) endometriosis had menstrual pain/cramping (52.7 vs. 45.2%, respectively) and non-menstrual pelvic pain/cramping (36.7 vs. 14.3%, respectively) [27]. In contrast to our findings, Ballard et al. [14] reported that gnawing and throbbing, but not cramping or pulling, are more often experienced by women with endometriosis, which might be due to the lower number of women (n = 149) analyzed.

The questionnaire PainDETECT to evaluate neuropathic back pain [21,22] was used in the present study. Although we found a slightly higher proportion of women with endometriosis compared to women without endometriosis experiencing neuropathic pain (4.2% vs. 1.1%, respectively), it did not reach statistical significance. In contrast, two other studies revealed higher numbers of neuropathic pain cases in endometriosis patients (40%) [18] and patients with CPP (26%), using PainDETECT and other methods [32]. However, it was recently shown that after hysterectomy with or without ovarian preservation, the reoperation rates were very low (0.4–1.4%) [33], suggesting that the main pain sources are the uterus and the lesions and not neuropathic pain. 

The average of the final score of the PainDETECT resulted in significantly higher values in women with endometriosis compared to women without endometriosis (8.5 ± 5.5 vs. 3.7 ± 4.8, respectively). A higher proportion of women with endometriosis experience higher current pain, stronger pain in the last 4 weeks, higher mean pain, pain in the lower abdomen, lumbar spine pain, pain in the hips/groins, and pain in the thighs/lower extremities. The dragging pain in the legs was also reported by Ballard et al. [14] in women with endometriosis. Our analysis of the pain course pattern revealed a ~3-fold higher proportion of women with endometriosis suffering from pain attacks with moderate pain in between compared to women without endometriosis.

Although we found many new parameters formerly not described for endometriosis, we could confirm that no single pain parameter reached significance as a prognostic factor, as recently summarized [2]. We also observed an increased likelihood of endometriosis with an increased number of symptoms present; women with endometriosis experienced on average ~2-fold more significant symptoms compared to women without endometriosis, similarly to Ballard et al. [8]. In another study, four significant variables (CPP, dyspareunia with visual analogue scale (VAS) ≥3, painful defecation, and acne) resulted in a predictive logistic model with high sensitivity (0.902) and specificity (0.750) [15]. However, the use of hormonal contraception was not specified, and the high discrepancy in the percentage (6% vs. 44%) between women with and without endometriosis might confound the study. Similarly, a recent questionnaire also mentioned hormonal contraception only as a parameter beyond many others [34].

The strength of our study is the extensive evaluation of many parameters and the fact that all women underwent physical examination, palpation, TVUS, and MRI in cases of deep infiltrating endometriosis. Although all clinical examinations cannot exclude some positive cases of endometriosis [35], with the questionnaire, we are reasonably sure that the women were not suffering from endometriosis-associated pain, and it is possible to re-analyze these cases with the questionnaire after some time. 

In our study, we generated a decision tree for early endometriosis prediction. Remarkably, the number of significant parameters was useful as the root of the tree. The decision tree contains dysuria, dyspareunia, dyschezia, obstipation, period pain, pain course pattern, pain radiating to the legs, pain in the lumbar spine, pain duration during menstruation, and the final score of the PainDETECT. The decision tree is divided into three main axes, suggesting that endometriosis is characterized by distinct pain patterns and not by single pain parameters. 

The decision tree analysis resulted in a high sensitivity of 0.904 and a specificity of 0.75, which is comparable with the sensitivity of 0.76 and specificity of 0.75 for bimanual examination, the sensitivity of 0.79 and specificity of 0.94 for TVUS, and the sensitivity of 0.94 and specificity of 0.77 for MRI, as recently summarized [36]. However, one has to keep in mind that TVUS and MRI are not very well suited for the detection of peritoneal endometriosis. In contrast, with our questionnaire, prediction of peritoneal, ovarian, and deep infiltrating endometriosis was possible with a sensitivity of 0.904 and a specificity of 0.75, which is very similar to the sensitivity of 0.94 and specificity of 0.79 postulated for a clinically useful non-invasive test for endometriosis [37].

### 4.1. Strengths and Weaknesses

In this study, we asked about almost all known pain parameters, so we assume that we have not missed too many. The questionnaire was optimized several times with statistical methods and thus validated. Furthermore, we also asked a sufficiently large number of patients. Up to date, however, the questionnaire has only been used in a clinical setting and only internally, not externally. 

### 4.2. Implications

The presented prediction model will not only enable the pre-operative and non-invasive diagnosis of endometriosis but can also be used by both patients and clinicians for surveillance of the disease before and after an operation. Similarly, patients without operations but with medical therapy can be monitored with the questionnaire. With the questionnaire, it will be possible to divide patients in advance into those who need immediate help and those who only need active monitoring. This could contribute to increased efficiency, saving time and money, and avoiding unnecessary operations.

## 5. Conclusions

In this study, we have shown that several distinct pain patterns are highly predictive of endometriosis. In contrast to women without endometriosis, women with endometriosis experience more pain, and a very high number of cases describe their pain as cramping, tearing, or pulling, which points to the uterus as the main pain contributor. Surprisingly, neuropathic pain was not very common in women with endometriosis and did not differ significantly from women without endometriosis. The present study clearly demonstrates that the pre-operative diagnosis of endometriosis based upon questionnaires is possible and underscores that we should ‘never underestimate the pain’ [38]. Thus, physicians should always consider endometriosis as a possible cause of severe period pain, which should be treated immediately.

## Figures and Tables

**Table 1:** Demographics of patients with and without endometriosis.

	No EM N = 92	MD	EM N = 177	MD	*p* Value
Age (yrs), mean (SD)	35.1 (8.8)	0	34.3 (6.7)	0	0.485
BMI, mean (SD)	24.2 (4.9)	1	24.2 (5.3)	0	0.921
Age Menarche, mean (SD)	13.1 (1.5)	2	13.0 (1.5)	2	0.698
Smoker (%)	24 (26.1)	0	52 (29.4)	0	0.669
Allergies (%)	43 (46.7)	0	106 (60.6)	2	0.038
Cycle duration in days (SD)	27.5 (5.1)	4	27.4 (5.5)	8	0.689
Menstruation duration in days (SD)	5.2 (1.9)	3	6.1 (4.0)	2	0.119
Irregular Cycle	28 (31.5)	3	55 (32.4)	7	1.0
Abnormal Menses	29 (34.1)	7	61 (36.7)	11	0.781
Use of analgesics (%)	47 (51.1)	0	143 (80.8)	0	0.0001
Analgesics before menses (%)	5 (13.2)	0	9 (7.4)	0	0.274
Analgesics during menses (%)	27 (71.0)	0	69 (56.6)	0	0.113
Analgesics before/during menses (%)	6 (15.8%)	0	42 (34.4%)	0	0.029
Analgesics before/during/after menses (%)	0 (0%)	0	2 (1.6%)	0	0.7
	Neg	Pos	Neg	Pos	
Nulliparous	39	0	54	0	
Fertility (%)	11 (20.8)	42 (79.2)	61 (49.6)	62 (50.4)	0.0004
Abortion (%)	40 (70.5)	13 (24.5)	87 (70.7)	36 (29.3)	0.714

EM, endometriosis; MD, missing data; BMI, body mass index; yrs, years; SD, standard deviation; Neg, negative; Pos, positive; *p* values calculated by Fisher’s exact test (smoker, allergies, irregular cycle, fertility, abortion) or Mann-Whitney (all the others) where appropriate.

**Table 2:** “Classical” pain parameters of patients with and without endometriosis.

	No EM N = 92		EM N = 177		*p* Value	MD
	No	Yes	No	Yes		
Dysmenorrhea (PP)	24	68	5	172	0.0001	0
PP, NRS ≤ 3	35	57	6	171	0.0001	0
PP, NRS score (SD)	5.1 (3.8)		7.8 (2.2)		0.0001	0
PP before menses (%)	8 (11.9)		12 (7.4)		0.304	
PP during menses (%)	22 (32.8)		28 (17.2)		0.013	
PP before/during menses (%)	27 (40.3)		58 (35.6)		0.549	
PP before/during/after menses (%)	8 (11.9)		65 (39.9)		0.0001	
PP after menses (%)	0 (0)		0 (0)		1.0	
PP during/after menses (%)	2 (3.0)		5 (3.1)		1.0	
PP before/after menses (%)	0 (0)		1 (.6)		1.0	
Chronic pelvic pain (CPP)	73	19	95	82	0.0001	0
CPP, NRS score (SD)	1.4 (2.9)		2.9 (3.4)		0.0001	0
Dysuria (painful urination)	88	4	120	57	0.0001	0
Dysuria, NRS score (SD)	0.22 (1.1)		1.63 (2.7)		0.0001	0
Dyschezia (painful defecation)	78	14	94	83	0.0001	0
Dyschezia (NRS ≤ 1)	79	13	94	83	0.0001	0
Dyschezia, NRS score (SD)	0.85 (2.2)		3.03 (3.5)		0.0001	0
Dyspareunia (painful intercourse)	59	26	72	104	0.0001	7/1
Dyspareunia (NRS ≤ 1)	60	25	72	104	0.0001	7/1
Dyspareunia, NRS score (SD)	1.5 (2.6)		3.5 (3.4)		0.0001	7/1
Obstipation	78	14	106	71	0.0001	0
Diarrhea	73	19	114	63	0.0122	0

EM, endometriosis; SD, standard deviation; MD, missing data 7/1 means 7 healthy women and 1 case of endometriosis; *p* values calculated by Fisher’s exact test or Mann-Whitney (NRS scores) where appropriate.

**Table 3:** Pain sensations of patients with and without endometriosis.

	No EM N = 92		EM N = 177		*p* Value
	No	Yes	No	Yes	
Cramping	45	47	39	138	0.0001
Tearing	77	15	105	72	0.0001
Pulling	52	40	53	124	0.0001
Stinging	58	34	76	101	0.0020
Pulsatile	81	11	136	41	0.0338
Touch-sensitivity	79	13	128	49	0.0143
Burning	86	6	148	29	0.0225
Pressing	86	6	154	23	0.1459
Diffuse	84	8	152	25	0.2420
Heat	87	5	161	16	0.3469
Flashing	82	10	154	23	0.6979

EM, endometriosis; no missing data found; *p* values were calculated by Fisher’s exact test.

**Table 4:** Pain characteristics and localization with the PainDETECT.

	No EM N = 92		EM N = 177		*p* Value
	Neg	Pos	Neg	Pos	
Pain now	68	24	88	89	0.0001
Pain now, NRS (SD)	1.07 (2.2)		2.0 (2.6)		0.0003
Strongest pain/4 wks	37	55	11	166	0.0001
Strongest pain, NRS (SD)	4.3 (4.0)		7.6 (2.7)		0.0001
Average pain	42	50	18	159	0.0001
Average pain, NRS (SD)	2.7 (2.9)		4.9 (2.7)		0.0001
Lower abdomen	40	52	18	159	0.0001
Lumbar spine pain	65	27	60	117	0.0001
Thighs/lower extremities	78	14	117	60	0.001
Hips/groins	85	7	144	33	0.018
Upper abdomen	89	3	159	18	0.055
Vagina/Mons pubis	81	11	152	25	0.708
Gluteal region	88	4	168	9	1.0
Pain course pattern	38	54	10	167	0.0001
- Persistent pain with slight fluctuations (%)		5 (9.3)		17 (10.2)	1.0
- Persistent pain with pain attacks (%)		22 (40.7)		50 (29.9)	0.186
- Pain attacks without pain between them (%)		24 (44.4)		72 (43.1)	0.241
- Pain attacks with pain between them (%)		3 (5.6)		28 (16.8)	0.0427
Final score (Neuropathic pain)					
- Neg 0–12 (%)	87 (94.6)		136 (76.8)		0.0001
- Unclear 13–18 (%)	4 (4.3)		33 (18.6)		0.0012
- Pos 19–38 (%)	1 (1.1)		8 (4.5)		0.1721
Final score, mean (SD)	3.7 (4.8)		8.5 (5.5)		0.0001
- 0 vs. 1–38	42	50	12	165	0.0001
- 0–3 vs. 4–38	55	37	37	140	0.0001

EM, endometriosis; SD, standard deviation; wks, weeks; no missing data; Neg, negative; Pos, positive; *p* values calculated by Fisher’s exact test or Mann-Whitney (NRS).

**Table 5:** Significant parameters and decision tree analysis.

	No EM N = 92		EM N = 177		*p* Value
	No	Yes	No	Yes	
**Significant parameters (SD)**	7.8 (5.8)		14.1 (4.2)		<0.0001
Cut-off of 8	46	46	12	165	<0.0001
Positive predictive value (95% CI)					0.782 (0.7203–0.8358)
Negative predictive value (95% CI)					0.7931 (0.6666–0.8881)
Sensitivity (95% CI)					0.9322 (0.8843–0.9645)
Specificity (95% CI)					0.5000 (0.3942–0.6058)
Odds ratio (95% CI)					13.75 (6.729–28.097)
Relative risk (95% CI)					3.78 (2.272–6.288)
**Decision tree**	69	23	17	160	<0.0001
Positive predictive value (95% CI)					0.8743 (0.8175–0.9187)
Negative predictive value (95% CI)					0.8023 (0.7028–0.8802)
Sensitivity (95% CI)					0.904 (0.8508–0.943)
Specificity (95% CI)					0.75 (0.6485–0.8341)
Odds ratio (95% CI)					28.235 (14.195–56.163)
Relative risk (95% CI)					4.423 (2.879–6.795)

EM, endometriosis; SD, standard deviation; *p* values calculated by Fisher’s exact test or Mann–Whitney, where appropriate.

## Data Availability

The data is available from the corresponding author upon request.

## Supplementary Materials

The following supporting information can be downloaded at: https://www.mdpi.com/article/10.3390/jcm12134231/s1.
