# Supplementary material for: Predictive Model for the Non-Invasive Diagnosis of Endometriosis Based on Clinical Parameters
Source: J Clin Med. 2023 Jun 23;12(13):4231. doi: 10.3390/jcm12134231 (PMC10342998; doi:10.3390/jcm12134231)
Supplement: Supplementary file 1 [file jcm-12-04231-s001.zip › jcm-2419838-supplementary.pdf]

Endometriosis-Questionnaire Date

Family name:______________________ First name:_______________________

Date of birth:________________

Height:______________________ Weight:_______________________

Is endometriosis known in your family? □ Yes □ No □ Don't know
If yes, who?
□ Grandmother
□ Mother
□ Sister
□ Aunt

Are gynecological cancers known in your family? □ Yes □ No □ Don't know
If yes, who?
□ Grandmother
□ Mother
□ Sister
□ Aunt

Are gastrointestinal cancers known in your family? □ Yes □ No □ Don't know
If yes, who?
□ Mother
□ Father
□ Sister
□ Brother
□ Grandmother
□ Grandfather
□ Aunt
□ Uncle

Are other cancers known in your family? □ Yes □ No □ Don't know
If yes, who?
□ Mother
□ Father
□ Sister
□ Brother
□ Grandmother
□ Grandfather
□ Aunt
□ Uncle

Are you smoking? □ Yes □ No
How many cigarettes daily? ________ Cigarettes
Since how many years? ________

Are you drinking alcohol?
□ No
□ sometimes
□ regularly
□ excessively

Are you suffering from allergies? □ Yes □ No □ Don't know
If yes, which?
□ Pain drugs
□ Antibiotics
□ Band-aid, nickel,
Other contact compounds
□ Hay fever
□ Others: _____________

Are chronic diseases or pre-existing illnesses known?<br>If yes, which?	□ Yes □ No □ Don't know<br>□ Asthma<br>□ Autoimmune diseases<br>□ Diabetes<br>□ Hypertension<br>□ Thyroid diseases<br>□ Others:______________
Are you working in shift work?<br>If yes, since when?	□ Yes □ No<br>____________
Have you already been diagnosed with endometriosis?<br>If yes, since when?	□ Yes □ No<br>________________________
Have you already been operated because of endometriosis?<br>If yes, in which year(s)?	□ Yes □ No<br>Abdominal sections:<br>Numbers:________________<br>Year(s):_________________<br><br>Laparoscopies:<br>Numbers:________________<br>Year(s):_________________<br><br>Vaginal__________________<br>Numbers:________________<br>Year(s):_________________
Have you been operated because of an ovarian cyst?<br>If yes, in which year(s)?	□ Yes □ No<br>Laparoscopies:<br>Numbers:________________<br>Year(s):_________________
Did you have another gynecologic pelvic operation in the past?<br>If yes, in which year(s)?	□ Yes □ No<br>Laparotomies:<br>Numbers:________________<br>Year(s)__________________<br><br>Laparoscopies:<br>Numbers:________________<br>Year(s)__________________<br><br>Vaginal:<br>Numbers________________<br>Year(s)__________________
Have you had an intestinal operation in the past?<br>If yes, in which year(s)?	□ Yes □ No<br>Laparotomies:<br>Numbers:________________<br>Year(s)__________________<br><br>Laparoscopies:<br>Numbers:________________<br>Year(s)__________________
Have you already had an operation on your pelvis for other reasons that have not yet been mentioned?<br>If yes, in which year(s)?	□ *Yes* □ *No*<br><br>Laparotomies:

Numbers:________________
Year(s)__________________

Laparoscopies:
Numbers:________________
Year(s)__________________

Have you had a curettage/erasion in the past? □ Yes □ No
If yes, in which year(s)?
Bleeding disorder:________
Diagnostic:_____________
Abortion:_______________

In which age have you had your first menstruation? _______________

In which age have you started hormonal contraception? _______________________

Is your cycle regular? □ Yes □ No
How many days is your cycle on average? ___________Days
How long is your bleeding on average? ___________Days
When was your last bleeding? ___________Date

How do you evaluate the bleeding intensity of your last menstruation?
□ unchanged
□ stronger
□ weaker than usual

Are you currently using? □ Yes □ No
If yes, how do you prevent pregnancy?
□ Oral contraception
□ IUD (intrauterine device)
□ Contraceptive coil (Mirena)
□ Vaginal ring (Nuvaring)
□ 3-month-depot (e.g.syringe)
□ Condom
□ Others: _______________

Since when do you use hormonal contraception regularly? _______________________

Which oral contraception are you using for the time being? _______________________
If yes, since when you are using it? ______________________

Have you been pregnant? □ Yes □ No
If yes, how often? __________

How have you become pregnant?
□ natural ___times
□ stimulated by hormones ___times
□ Insemination ___times
□ IVF ___times
□ ICSI ___times
□ others ___times
□ I don't know

Have you received an artificial fertilization? □ Yes □ No
Was it successful? □ Yes □ No

How were the children born?
Natural, which year(s)? ___________
By caesarean section, which year(s)? ___________
Vaginal operation (suction cup etc.), which year (s)? __________

Did you have a curettage after the birth of your child? □ Yes □ No
If yes, in which year(s)? _________
Why was the curettage performed? □ Placenta dissolution disturbance
□ others:_____________

Are you currently planning to have children? □ Yes □ No

Are you involuntarily childless? □ Yes □ No
If yes, since when? __________

Has your partner had a fertility test related to this? □ Yes □ No

If yes, what was the result? □ without finding
□ impaired

How long have you wanted to have children with regular sexual intercourse? ____________

Do you have pain related to your period? □ Yes □ No
If yes, when? □ before
□ during
□ after

How bad is the period pain on a scale of 1 to 10?
No pain □ □ □ □ □ □ □ □ □ □ strongest pain
1 2 3 4 5 6 7 8 9 10

Do you have chronic pain in the lower abdomen? □ Yes □ No
If yes, how bad is your pain on a scale from 1 bis 10?
No pain □ □ □ □ □ □ □ □ □ □ strongest pain
1 2 3 4 5 6 7 8 9 10

Do you have pain when urinating? □ Yes □ No
If yes, how bad is your pain on a scale from 1 bis 10?
No pain □ □ □ □ □ □ □ □ □ □ strongest pain
1 2 3 4 5 6 7 8 9 10

Do you have pain during defecation? □ Yes □ No
If yes, how bad is your pain on a scale from 1 bis 10?
No pain □ □ □ □ □ □ □ □ □ □ strongest pain
1 2 3 4 5 6 7 8 9 10

Do you feel pain during sexual intercourse? □ Yes □ No
If yes, how bad is your pain on a scale from 1 bis 10?
No pain □ □ □ □ □ □ □ □ □ □ strongest pain
1 2 3 4 5 6 7 8 9 10

Do you take painkillers? □ Yes □ No

If yes, which ones? ________________ ________________

If yes, which amount(s)? ________________

If yes, since when? ________________

If yes, when do you take the painkillers? □ Regularly
□ if necessary

□ before the period (start)
□ during the period

Can you describe your pain? (burning, stinging, diffuse, heat-sensitive, touch-sensitive, pulsatile, cramping, pulling tearing etc.) ________________

Do you have frequent constipation? □ Yes □ No
If yes, how often, how long? ________________

Do you have frequent diarrhea? □ Yes □ No
If yes, how often, how long? ________________

Have you ever noticed blood deposits on your stool? □ Yes □ No

Have you ever received hormonal treatment for your endometriosis? □ Yes □ No □ Don't know
If yes, which treatment did you receive in which year?

Oral contraception:_______
GnRH: ____________
Hormonal IUD: ____________
others: ____________
